# New mathematical model based on geometric algebra for physical power flow in theoretical two-dimensional multi-phase power circuits

**DOI:** 10.1038/s41598-023-28052-x

**Published:** 2023-01-20

**Authors:** Francisco G. Montoya, Xabier Prado, Francisco M. Arrabal-Campos, Alfredo Alcayde, Jorge Mira

**Affiliations:** 1 Department of Engineering, University of Almeria, 04120 Almeria, Spain; 2 Departamento de Física Aplicada and iMATUS, Universidade de Santiago de Compostela, 15782 Santiago de Compostela, Spain

**Keywords:** Electrical and electronic engineering, Applied physics

## Abstract

This study proposes an explanation for the physical power flow in planar circuits by analogy to theoretical two-dimensional circuits using a new mathematical model based on Geometric Algebra (GA) and 2D Maxwell’s equations. In contrast with traditional 3D physics in the observable real world, the magnetic field can be defined as a bivector instead of an axial vector allowing to obtain the Poynting Vector directly in a 2D flat world, where physical variables of planar circuits can be obtained. This approach is presented here for the first time to the best of the author’s knowledge. Previous investigations have focused on simplifications and symmetries of real 3D circuits studied mainly in the phasor and frequency domain. In this work, the electromagnetic power flow phenomenon is analyzed on a completely 2D time-domain basis and derived directly from the undisputed Maxwell equations, formulated in two dimensions. Several cases of special interest in AC multi-phase circuits are presented using the proposed technique, bringing a new simplified approach to the measurement of power flow exchange between the source and the load. It suggests a new way to understand energy propagation from a purely physical point of view.

## Introduction

### Motivation

Modelling physical variables in multi-phase systems in general, and three-phase in particular is of vital importance for the efficient operation of power grids. In this respect, non-sinusoidal or unbalanced circuits in new-generation smart grids are of great interest today because they operate under sub-optimal conditions, leading to higher energy losses in distribution and transmission lines. Therefore, the determination of the physical conditions for the power to flow in a non-symmetrical situation is of great relevance^1^. Moreover, the optimization of such parameters is also of paramount importance because of the new grid model, greatly influenced by renewable power sources^2^. This work proposes new insights to deal with power flow in simplified 2-dimensional scenarios that can be generalized to realistic 3-dimensional ones through the theoretical framework provided by Geometric Algebra (GA).

### Background and literature review

Previous investigations on power properties^3–5^ have been based on concepts related to frequency and phasors (complex algebra) using non-physical definitions such as apparent power *S* or non-active power *N*. They may be relevant from an economic point of view, but they are not justified from a purely physical perspective, and therefore, they cannot be measured directly. This aspect has been the source of a long and heated controversy that still survives in the electrical engineering community. A recent paper by some of the authors favours the physical time-based domain approach to shed light on this debate^6^. In the view of the authors, definitions based on non-physical concepts are the real underlying problem that has failed to address this century-old problem. Therefore, rather than non-realistic definitions, we suggest a framework based on physical variables, which are measurable by means of appropriate instrumentation: voltage/current, electric/magnetic field and power/energy.

The most physically and meaningful measurable quantity is the instantaneous power *p*(*t*). Unfortunately, *p*(*t*) (measured at the load terminals) provides a simplified and incomplete representation of the picture. The most widely known example is that of a balanced three-phase system with a symmetrical supply, where the value for *p*(*t*) is constant even though each phase demands a fluctuating power separately. This in turn leads to a paradox widely debated^7–9^ concerning the impossibility of obtaining the reactive power *Q* and therefore the inability of providing a measure of the system efficiency concerning the power losses in the transmission lines (i.e. the power factor). Consequently, the stored/restored energy of the reactive elements cannot be measured either.

Some authors have tried to find a convincing explanation for the balanced and unbalanced conditions by making use of the Poynting Vector (PV)^10^, obtaining interesting results for simple circuits^11,12^. In a paper of Todeschini et al^13^, the Steinmetz compensator is analyzed and a very interesting conclusion is drawn: an electric power exists fluctuating in the surrounding space around the conductors that do not contribute to a net transfer of energy for the load. For this purpose, the authors present an example of a transmission line consisting of very long, two-dimensional sheet-shaped cables separated by a very small distance. In this 3D geometry, it is easy to compute the magnetic and electric fields because this scenario is an equivalent way of working in a two-dimensional system. This is not the case for other general arbitrary 3D configurations.

Therefore, the PV has proven to be useful^14^ because it takes into account the specific phenomena during power transmission through space and not only at the load terminals. However, it presents two major challenges. On the one hand, by combining two physical quantities (electric and magnetic fields), the degree of complexity of the signal represented by each of them is increased. On the other hand, since it is a spatially distributed vector field, the difficulty derived from the specific geometry of each circuit is inherited.

### Contributions

To overcome the abovementioned challenges, we propose to solve the power flow problem in a new theoretical 2D (flat) world. In this flat world, it is possible to escape the 3D geometrical sophistication to focus on the complexity of the signals^15^. This is particularly interesting when considering multi-phase circuits that may be unbalanced or distorted. This approach will reveal the PV as an effective tool to study and analyze re-balancing solutions. We explicitly stress the fact that no third dimension exists in our 2D flat world. The novel contributions are the following:

- A new and different approach to power flow through electromagnetic principles in a two-dimensional world is proposed using a new mathematical framework based on Geometric Algebra as a unifying and enabling tool in engineering.
- As a result, it is possible to present some fundamental laws in a synthetic form that can be directly applied to pure two-dimensional lumped parameter circuits with minimal adaptations. Therefore, we claim that our work also makes a contribution towards the unification of circuit theory and field analysis approach going a step further in our previous investigations^16^.
- A relevant novelty is to consider the magnetic field as a bivector (rather than an axial vector). Note that the vector product can only be computed in 3D. In this regard, the traditional definition of PV corresponds to the vector product of the electric and magnetic field, i.e. $\vec {E}\times \vec {H}$. However, the vector product is not mathematically defined for any dimension different than 3 (or 7 depending on the chosen definition), so it cannot be applied in a flat world. In this way, the proposed GA-based PV can be applied to purely planar circuits for the first time to the best of the author’s knowledge. Note that two-dimensional circuits are not a simple mathematical oddity. They are used in our work to facilitate problem understanding by considering the simplified geometries of a 2D world.
- The approach presented here also has some educational implications, allowing a better understanding of the power flow phenomena in a simplified scenario.

### Organization

The remainder of the paper is organized as follows: Sect. “2D spatial power flow and poynting vector in the geometric algebra formalism” describes the spatial power flow in 2D using GA and PV; Sect. “Applications and examples” presents the empirical validation through different applications and examples; Sect. “Conclusions” provides the main conclusions obtained.

## 2D spatial power flow and poynting vector in the geometric algebra formalism

The aim here is to investigate the allocation of power flow between a load and a source defined entirely in a theoretical 2D flat world. Even though this hypothesis may seem to have no practical justification, it can help to better understand the power flow mechanism in the traditional lumped-parameter circuits typically used in electrical engineering, where it is common to overlook the spatial component in favour of a more simplified model at the cost of losing some physical intuition on how the power flows and, thus, reaches the load.

Physical reality we all know is spatially three-dimensional (3D)^17^, but in cases where symmetries are presented in one direction, it is worth considering alternative approaches. For example, the use of a lower dimensional framework can lead to a reduction in the complexity of the problem. This approach is widely used in several branches of engineering as in mechanics^18^. In principle, one might think that a limit to this modus operandi is the need for a description of magnitudes involving the cross product. Electrical engineering is an area where this happens^19^. Nevertheless, this issue can be avoided thanks to Geometric Algebra (GA). One of the cornerstones of this exercise is the use of the concept of bivector, a new mathematical 2D object that can be used to represent a plane. It can be built by wedging two vectors and the result is an object with direction, sense and magnitude similar to a vector. The other one is the geometric product, a bilinear operation that results in a multivector, which is a generalization of the vector concept. Armed with these tools, it is possible to waive the cross product and develop a 2D formulation of electromagnetism^16^. GA encompasses and generalizes many of the different mathematical tools and concepts used in various fields, including differential forms, quaternions, complex numbers, tensors or matrices. It is a powerful tool that can provide a more intuitive and efficient way to describe and analyse physical phenomena, including Maxwell’s equations^20^. One of the main benefits of using GA is that it allows for the compact representation of complex mathematical operations using a simple algebraic language. This can make it easier to understand and manipulate the equations, as well as identify and exploit any symmetries or conservation laws that may be present. It also has the ability to naturally represent and manipulate objects and operations in higher and lower dimensions (different from our 3D world). This allows for the representation of complex geometric objects, such as planes and spheres, as well as operations such as rotations and translations, in any number of dimensions. Note that other approaches exist for defining the PV, such as those based on the use of space-time tensors and Noether’s theorem^21^. However, they are not the most suitable and adequate for working with electrical circuits, thus the use of GA is preferred instead due to its greater simplicity and convenience in the field of electrical engineering as it has been shown in recent works^22–24^.

Moreover, unlike previous contributions found in the literature, it will not be necessary to make use of definitions based on complex algebra such as the apparent or non-active power, which is widely discussed and disputed by the community^25,26^. As will be seen, the PV will play a fundamental role in this process.

### Electric and magnetic field in 2D using geometric algebra

Recently, Geometric Algebra has been successfully applied in physics^27,28^ and electrical engineering^6,22,29–31^. It enables a reformulation of electromagnetic Maxwell’s equations in a very compact and unified form^28^. It also generalises other approaches previously conducted in the literature, such as differential forms, allowing to keep pace with the extended vector nomenclature for the laws of electromagnetism and providing advantages from a pedagogical and conceptual standpoint^32,33^. Thus, Maxwell’s equations reduce to a single expression (see Appendix B)

1 $$\begin{aligned} \nabla \varvec{F} = \varvec{J} \end{aligned}$$

where $\varvec{F}$ is the electromagnetic field multivector and $\varvec{J}$ is the sum of the density current vector ($\varvec{j}$) and free charges ($\rho$). As stated in^16^, it can be expanded to the following

2 $$\begin{aligned} \left( \partial _{t}+\varvec{\nabla }\right) ({\varvec{e}}+{\varvec{H}})=\rho -{\varvec{j}} \end{aligned}$$

where $\varvec{H}$ is the magnetic field. Note that we use the letter $\varvec{e}$ for the electric field instead of the traditional $\varvec{E}$ because, in the rest of this document, lowercase bold letters are reserved for vectors while uppercase bold letters are used for multivectors. Scalars are represented using non-bold letters or symbols. The nabla operator in bold is defined as a vector $\varvec{\nabla }_{}=\sum _{i=1}^k \partial _{i} \varvec{\sigma }_{i}$ with *k* the number of dimensions. Note the use of an Euclidean orthonormal basis $\varvec{\sigma }=\{\varvec{\sigma }_1, \varvec{\sigma }_2,\ldots , \varvec{\sigma }_k\}$. Equation (2) can be applied to spaces of dimension other than three. More precisely, for a two-dimensional space, the quantities involved can be described in their components (for clarity) *x* and *y* as follows:

3 $$\begin{aligned} \begin{aligned} {\varvec{j}}&=j_{x} \varvec{\sigma }_{x}+j_{y} \varvec{\sigma }_{y} \\ {\varvec{e}}&=e_{x} \varvec{\sigma }_{x}+e_{y} \varvec{\sigma }_{y} \\ {\varvec{H}}&=H \varvec{\sigma }_{x y} \end{aligned} \end{aligned}$$

One of the consequences of the application of GA is to obtain a magnetic field for two dimensions represented by a bivector (see Appendix A and B), something that, in the usual vector algebra approach (Gibbs/Heaviside formalism) is not possible to compute or, at least, must be accomplished in an extremely unorthodox way^34,35^. In 2D, equation (2) can be expanded to three equations:

4 $$\begin{aligned} \begin{aligned} \varvec{\nabla } \cdot {\varvec{e}}&=\rho \quad&\text { (Gauss's Law) } \\ \varvec{\nabla } {\varvec{H}}&=-{\varvec{j}}-\partial _{t} {\varvec{e}} \quad&\text { (Amp}\grave{\textrm{e}}\text {re's Law) } \\ \varvec{\nabla } \wedge {\varvec{e}}&=-\partial _{t} {\varvec{H}} \quad&\text { (Faraday's Law) } \end{aligned} \end{aligned}$$

**Figure 1:**
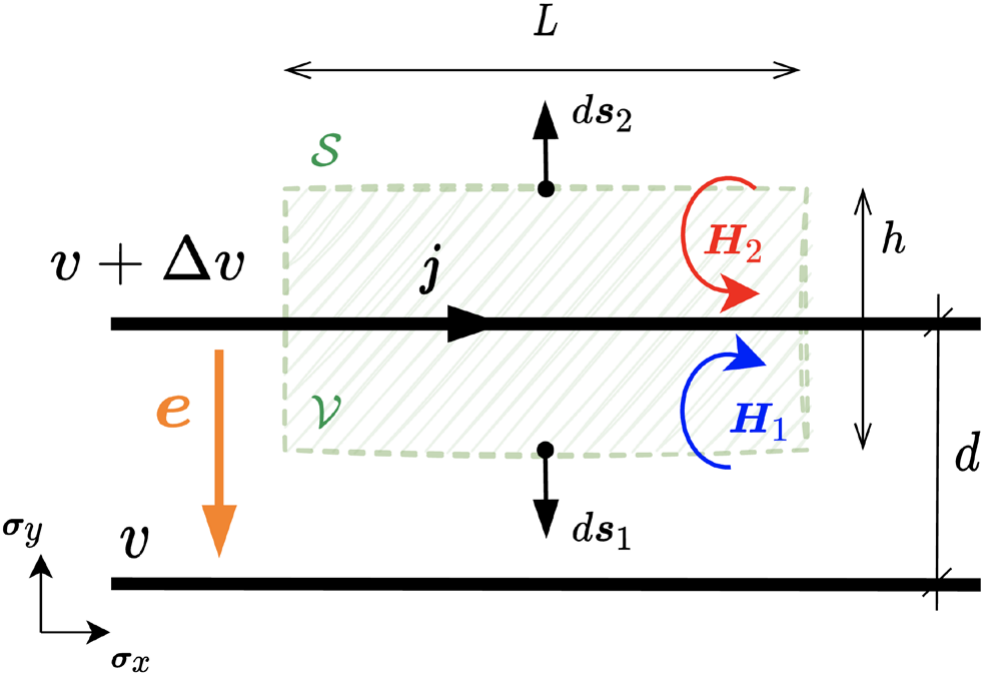
Relevant variables for the computation of magnetic and electric fields for two long parallel wires. Only the current on the top cable has been considered. The orientation for $\varvec{e}$ and $\varvec{H}$ follows positive values of the fields.

A detailed derivation of the above equations using coordinates is shown in Appendix B. In the present work, we are interested in the calculation of the values of $\varvec{e}$ and $\varvec{H}$ for the electroquasistatic (EQS) and magnetoquasistatic (MQS) conditions from the line potentials (*v*) and currents (*i*) for indefinitely long parallel wires, representing transmission or distribution power systems as depicted in Fig. 1. The EQS and MQS follow based on the assumption that the wavelength of the signals involved (with frequency in the range of a few kHz maximum) is much higher than the *d* parameter. This is a reasonable assumption for typical power systems, i.e., lines and circuits ranging from centimetres to a few kilometres. For the calculation of $\varvec{e}$, the usual expression as a function of the potential difference $\Delta v$ existing between a pair of parallel perfect conducting wires separated by a distance *d* will be used. The rationale is that, in two dimensions, the value of the field $\varvec{e}$ depends only on the two nearest wires to the point under consideration (note that no longitudinal component of $\varvec{e}$ is considered in this case). It can be readily computed as

5 $$\begin{aligned} \varvec{e}=-\varvec{\nabla }v=-\frac{\Delta v}{d} \varvec{\sigma }_{y} \end{aligned}$$

Notice that the $\varvec{e}$ is always perpendicular to the direction of the wires and its orientation follows from (5). In addition, the principle of superposition must be applied to calculate $\varvec{H}$ so that the effects of all existing currents can be taken into account. For typical conductive circuits, the displacement current ${\varvec{j}}_{D}=\partial _{t} {\varvec{e}}$ can be neglected under quasi-static condition^36^. For the circuits analyzed here, this condition holds because the wavelength at the frequency of interest (50/60 Hz and harmonics up to a few thousand Hz) is much longer than the length scales of the circuits^37,38^. In this case, Ampère’s law reduces to:

6 $$\begin{aligned} \begin{aligned} \varvec{\nabla } {\varvec{H}}&=\varvec{\nabla } \cdot {\varvec{H}}={\varvec{j}} \\ \int _{{\mathscr {V}}}^{} \varvec{\nabla } {\varvec{H}} d \varvec{\tau }&=\oint _{{\mathscr {S}}}^{} {\varvec{H}} \cdot d s=\int _{{\mathscr {V}}}^{} \varvec{j} d \varvec{\tau } \end{aligned} \end{aligned}$$

Note that the fundamental theorem of calculus has been used in the above expression (see Appendix B). Note also that, for a two-dimensional space, the hyper-volume ${\mathscr {V}}$ is a surface delimited by the rectangle ${\mathscr {S}}$ with length *L* and height *h* (see Fig. 1).

The integral for the density current $\varvec{j}$ in the hyper-volume ${\mathscr {V}}$ can be computed easily as:

7 $$\begin{aligned} \int _{{\mathscr {V}}}^{} \varvec{j} d \varvec{\tau }=L \varvec{j} = Lj\varvec{\sigma }_x \end{aligned}$$

For the integral over the boundary ${\mathscr {S}}$, the contributions to $\varvec{H}\cdot d\varvec{s}$ on the sides of height *h* cancel each other out because of the symmetry of the system, while on the sides of length *L* their value is:

8 $$\begin{aligned} \begin{aligned}{}&{\varvec{H}}_{1} \cdot d {\varvec{s}}_{1}=-H \varvec{\sigma }_{x y}\left( - ds \varvec{\sigma }_{y}\right) =H ds \varvec{\sigma }_{x} \\&{\varvec{H}}_{2} \cdot d {\varvec{s}}_{2}=H \varvec{\sigma }_{x y}\left( ds \varvec{\sigma }_{y}\right) =H ds \varvec{\sigma }_{x} \end{aligned} \end{aligned}$$

so the line integral in (6) becomes

9 $$\begin{aligned} \oint _{{\mathscr {S}}}^{} {\varvec{H}} \cdot d \varvec{s}=2 H L\varvec{\sigma }_x \end{aligned}$$

We can obtain the magnitude of the magnetic field as $H=j/2$ by comparing expressions (6) and (7). Since the magnetic field is represented by a bivector, its orientation is represented using the notion of spin, as shown in Fig. 1. Note that this orientation is opposite on both sides of the wire carrying the density current $\varvec{j}$. We highlight that the above procedure has been carried out only for the current of the upper wire for simplicity, but it can be readily applied to the lower wire due to the superposition principle.

Once we have the magnetic and electric fields, the PV can be computed as

10 $$\begin{aligned} {\varvec{s}}={\varvec{H}}\cdot {\varvec{e}}=\varvec{He} \end{aligned}$$

Note that ${\varvec{H}}\wedge {\varvec{e}}=0$ since the electric field vector lies in the same plane as the magnetic bivector. Therefore, the PV can be defined as the geometric product of ${\varvec{H}}$ and ${\varvec{e}}$. This fact has relevant implications to compute the electric or magnetic field from the product between their corresponding inverses and the PV.

**Figure 2:**
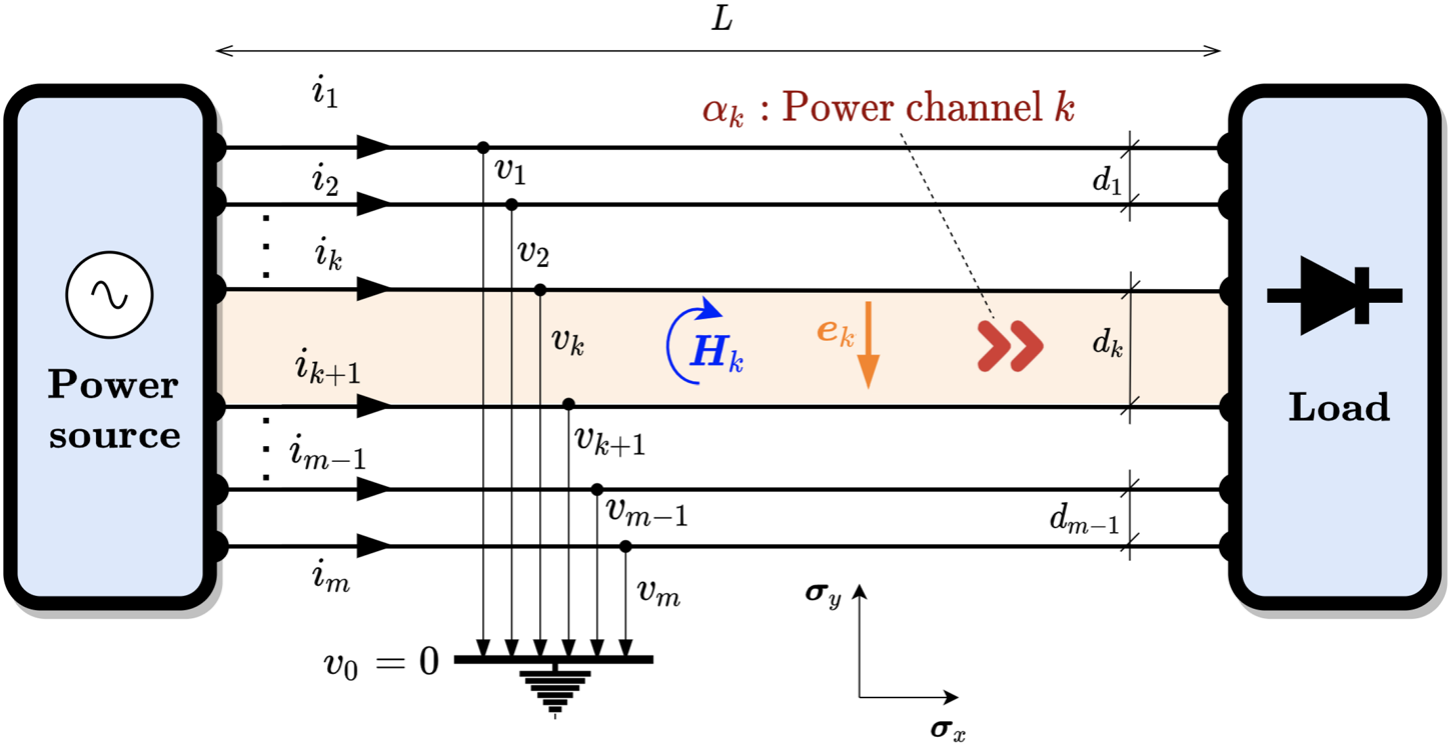
Generic multi-phase circuit with *m* wires. The power flows through every power channel $\alpha _k$ delimited by wires *k* and $k+1$.

### Poynting vector and powers in 2D for multi-phase circuits

The system in Fig. 2 represents a multi-phase *m*-wire 2D circuit with a voltage source and a generic load. Although it may look like a typical circuit with lumped parameters, in this case, the spatial geometry of the elements has been retained. It should be emphasized that our circuit lives in a 2 flat world, so there is no third dimension. The load and the source are native 2D elements in this theoretical world. This model presented hereby is valid for a hypothetical two-dimensional world but also to mimic certain realistic 3D configurations with appropriate symmetries or dimensions. We can regard Fig. 2 as the projection on the *xy*-plane of a 3D circuit made by conductor sheets with an extra *z*-dimension which extends indefinitely in comparison to the circuit’s measures, focusing the analysis only on points far away from the edges to avoid field and energy losses due to fringe effects. Alternatively, we can address circuits with a planar shape where the fields and energies are confined to a region surrounding the plane, such as in planar waveguides.

For now, we are only interested in the power flowing through the lines (very long), so we will not go into the detail of the load or source internals. The load is at a distance *L* from the source and the wires are separated by a distance $d_k$. The voltage of each line is referred to as a point considered at infinity (concerning the wires) with value $v_0=0$ V (virtual neutral) for convenience, however, any wire can be considered as a reference^39^ without changes in the power flow mechanism. We can see in the figure the presence of $m-1$ regions. As we shall see below, it is through these regions or channels that electrical power is transmitted and, hence, will be called *power channels*. They will designated by $\alpha _{k}$ with $k=1,2,\ldots ,m-1$. Again, we emphasize that power channels are purely 2D flat regions.

Using the derivations of Ampère’s and Gauss’s law from Sect. “Electric and magnetic field in 2D using Geometric Algebra”, it is possible to obtain information about the power transfer process from the source to the load through the power channel $\alpha _k$. To this end, it is necessary to make use of the PV defined in GA terminology. The electric and magnetic field for channel $\alpha _k$ is as follows

11 $$\begin{aligned} \begin{aligned} \varvec{e}_k&=\frac{v_{k+1}-v_{k}}{d_k}\varvec{\sigma }_y\\ \varvec{H}_k&=\frac{1}{2}\left[ \sum _{l=1}^k i_l-\sum _{l=k+1}^m i_l\right] \varvec{\sigma }_{yx}=\sum _{l=1}^ki_l\varvec{\sigma }_{yx} \end{aligned} \end{aligned}$$

Note that Kirchhoff Current Law (KCL) has been used in the second equation of (11). Note also that the magnetic and electric field is null outside the channels $\alpha _k$. The PV can be computed for every channel using equation (10), i.e., ${\varvec{s}}_{\alpha _k}={\varvec{H}}_k{\varvec{e}}_k$. By definition, the instantaneous power entering the load through the channel $\alpha _k$ is

12 $$\begin{aligned} \begin{aligned} p_{\alpha _k}&={\varvec{s}}_{\alpha _k}\cdot d_k\varvec{\sigma }_x=(v_k-v_{k+1})\sum _{l=1}^{k}i_l \end{aligned} \end{aligned}$$

and the total instantaneous power demanded by the load can be computed as

13 $$\begin{aligned} p=\sum _{k=1}^{m-1} p_{\alpha _k}=\sum _{k=1}^{m}v_k i_k \end{aligned}$$

The above result is completely general and matches the traditional expression for the instantaneous power computed using only the measured values of current and voltage at the load terminals. However, the benefit of introducing the power channels $\alpha _k$ through the PV is the ability to understand how the power enters the load from a spatial point of view. In the following sections, this fact will be revealed to understand the causes of the apparent paradoxes that might occur for the power flow under non-sinusoidal and unbalanced conditions.

## Applications and examples

Several examples and use cases of particular interest in multi-phase systems are presented below. For simplicity purposes, we mostly analyze pure resistive circuits, but any *RLC* combination can be also studied without major problems. The proposed method is successfully applied and compared to the result already known for the instantaneous power.

### Three-phase and three-wire balanced load and symmetric voltage supply

The first case of interest is the most basic one: a balanced three-phase and three-wire system supplied by a symmetric and sinusoidal voltage. Despite its simplicity, we believe that new methods should be tested against simplistic examples to demonstrate their validity. Therefore, we will use the results obtained in Sect. “Poynting vector and powers in 2D for multi-phase circuits” considering that $m=3$. The line to neutral voltages are

14 $$\begin{aligned} \begin{aligned} v_a(t)&=\sqrt{2}V\cos (\omega t)\\ v_b(t)&=\sqrt{2}V\cos (\omega t -120^{\circ })\\ v_c(t)&=\sqrt{2}V\cos (\omega t +120^{\circ })\\ \end{aligned} \end{aligned}$$

while the currents are

15 $$\begin{aligned} \begin{aligned} i_a(t)&=\sqrt{2}I\cos (\omega t + \varphi )\\ i_b(t)&=\sqrt{2}I\cos (\omega t + \varphi -120^{\circ })\\ i_c(t)&=\sqrt{2}I\cos (\omega t + \varphi +120^{\circ })\\ \end{aligned} \end{aligned}$$

From Fig. 2, we see that two power channels, i.e., $\alpha _1$ and $\alpha _2$, are created for this particular case. The power flowing through them can be calculated by applying (12)

16 $$\begin{aligned} \begin{aligned} p_{\alpha _1}&= \frac{v_{ab}}{2}(i_a-i_b-i_c)=v_{ab}i_a\\ p_{\alpha _2}&= \frac{v_{bc}}{2}(i_a+i_b-i_c)=v_{bc}(i_a+i_b)=v_{cb}i_{c} \end{aligned} \end{aligned}$$

Note that the total instantaneous power is obtained as $p=p_{\alpha _1}+p_{\alpha _2}=v_{ab}i_a+v_{cb}i_{c}$, which is, indeed, the Blondel-Aron theorem for three-phase three-wire systems, i.e. the two-wattmeter method. Note that this derivation is made from a purely physical point of view without any algebraic manipulation of the voltages and currents. Therefore, it is suggested that each wattmeter in the Blondel-Aron method measures the instantaneous power per channel. Substituting the values of the current and voltage given in (14) and (15) in (16), we get

17 $$\begin{aligned} \begin{aligned} p_{\alpha _1}&=\sqrt{3}VI\left[ \cos (2\omega t + \varphi + 30^{\circ })+\cos (\varphi - 30^{\circ })\right] \\ p_{\alpha _2}&=\sqrt{3}VI\left[ -\cos (2\omega t + \varphi + 30^{\circ })+\cos (\varphi + 30^{\circ })\right] \end{aligned} \end{aligned}$$

resulting in the well-known expression for the total instantaneous power, i.e., the active power

18 $$\begin{aligned} p=P=p_{\alpha _1}+p_{\alpha _2}=3VI\cos \varphi \end{aligned}$$

Moreover, the traditional reactive power can be computed as

19 $$\begin{aligned} \begin{aligned} Q&=\sqrt{3}({\bar{p}}_{\alpha _1}-{\bar{p}}_{\alpha _2})={3}VI\sin \varphi \\ \end{aligned} \end{aligned}$$

where ${\bar{p}}_{\alpha _k}$ is the mean value of the instantaneous power in channel $\alpha _k$. By analyzing Eqs. (17), we can observe that there is an oscillating power in each channel with an identical magnitude but of opposite sign, so they cancel each other resulting in a constant net power flow over time for the load. Therefore, thanks to the use of PV and the concept of 2D power channels, we can verify that there is a real oscillation of energy between the source and the load through each channel and that it cannot be perceived through its computation via the voltage and current at the terminals. In balanced systems, these oscillations are annihilated as a whole, so it can be stated that the symmetry in the current requires the power channels to present oscillations in phase opposition. As shown in subsequent sections, this is not the case for unbalanced systems.

Two interesting special cases can be considered depending on the value of $\varphi$ in (17), i.e., $\varphi =90^{\circ }$ (capacitive load) or $\varphi =-90^{\circ }$ (inductive load). For the capacitive case, we have

20 $$\begin{aligned} \begin{aligned} p_{C\alpha _1}&=VI\left[ \cos (2\omega t + 30^{\circ })+\frac{1}{2}\right] \\ p_{C\alpha _2}&=VI\left[ -\cos (2\omega t + 30^{\circ })-\frac{1}{2}\right] \end{aligned} \end{aligned}$$

and for the inductive case

21 $$\begin{aligned} \begin{aligned} p_{L\alpha _1}&=VI\left[ \cos (2\omega t + 30^{\circ })-\frac{1}{2}\right] \\ p_{L\alpha _2}&=VI\left[ -\cos (2\omega t + 30^{\circ })+\frac{1}{2}\right] \end{aligned} \end{aligned}$$

We can observe that each channel has a non-zero average power even though the sum of the channels results in a zero average power as expected. What is interesting here is to note that there is a sustained and directed non-zero mean power flow on each channel between the load and the source despite having purely reactive elements. This result seems quite interesting and challenges prior knowledge.

**Figure 3:**
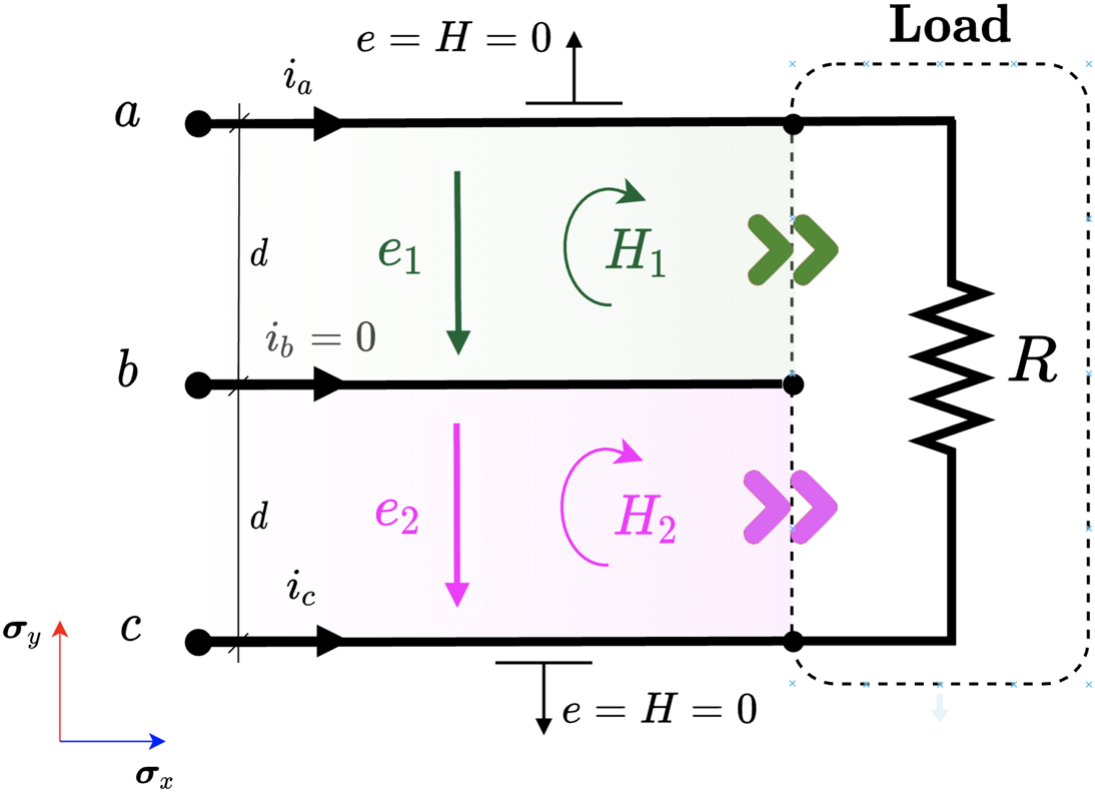
Magnetic and electric field in a 2D circuit with unbalanced three-phase load and symmetrical supply.

### Three-phase and three-wire unbalanced resistor load and symmetric voltage supply

An extremely unbalanced circuit is depicted in Fig. 3. This example has already been studied by Steinmetz and recently by other authors^13^. Notice that one of the phases is open (phase *b*) so that there is only current flowing from phase *a* to *c*. The traditional power analysis gives the following result at the load terminals,

22 $$\begin{aligned} \begin{aligned} p(t)&=v_ai_a+v_bi_b+v_ci_c=v_{ab}i_a+v_{cb}i_c\\&=\frac{v_{ac}^2}{R}=Ri_a^2 \end{aligned} \end{aligned}$$

The above expression is essentially Joule’s law applied to the resistor *R*. Note that the above voltages and currents can be arbitrarily time-dependent, although sinusoidal voltages and currents are of more engineering relevance. Therefore, we will use the sinusoidal and symmetrical voltage given in (14). The currents are easily obtained by applying Ohm’s law $i_a(t)=-i_c(t)=v_{ac}(t)/R$. The instantaneous power computed at the terminals of the load is

23 $$\begin{aligned} \begin{aligned} p(t)&=v_{ac}(t)i_a(t)=\frac{3V^2}{R} \left[ \cos (2\omega t + 60^{\circ })+1\right] \end{aligned} \end{aligned}$$

The above expression contains an oscillating term ($p_f$) and a constant term corresponding to the active power *P*. However, again, there is no information available on how the power flows before it enters the load. To determine this, we must turn to the PV. By substituting the line voltage values back into equations (12) and (13), the instantaneous power entering the load through channels $\alpha _1$ and $\alpha _2$ is obtained,

24 $$\begin{aligned} \begin{aligned} p_1(t)&=\frac{3V^2}{R} \left[ \cos (2 \omega t ) + \frac{1}{2}\right] \\ p_2(t)&=\frac{3V^2}{R} \left[ \cos (2 \omega t - 120^{\circ }) + \frac{1}{2}\right] \end{aligned} \end{aligned}$$

It is interesting to note that now the oscillating power flowing through each channel no longer has the same magnitude for each time instant (the same waveform is observed, but out of phase $120^{\circ }$). On the other hand, the active power *P* is equally distributed in each channel.

**Figure 4:**
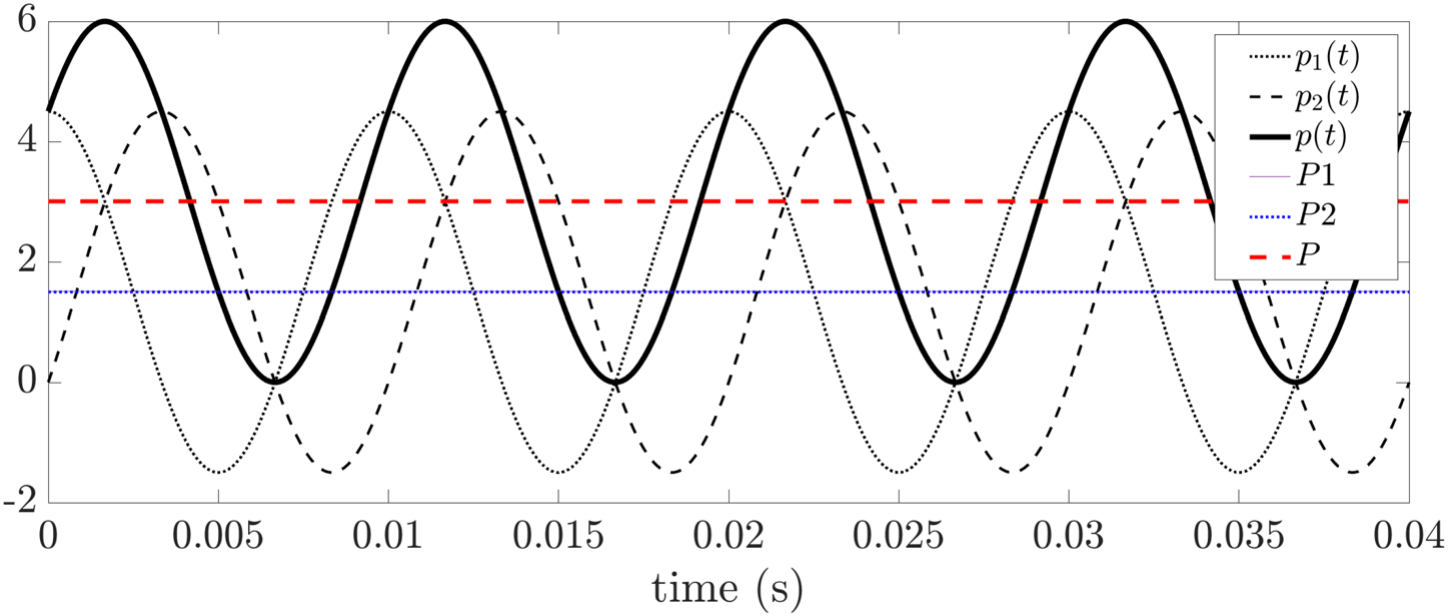
Power flow in channels $\alpha _1$ and $\alpha _2$, total instantaneous power and active power for the circuit in Fig. 3.

One may wonder how these channels may be formed since no current is flowing through line *b*. The explanation is simple. Although no current flows through *b*, the voltage of phase *b* is not null and therefore it affects the electric field $\varvec{e}_1$ and $\varvec{e}_2$, and hence the PV of the channels $\alpha _1$ and $\alpha _2$. Interestingly enough, line *b* is some kind of “electrical wall”.

Figure 4 shows the results for the values $V=1$ V, $f=50$ Hz and $R=1$ $\Omega$. It can be verified that the active power (average power) of each channel is the same ($P_1=P_2=1.5$ W) and, of course, they add up to the total active power expected in the resistor *R* calculated by Joule’s law ($P=3$ W). The most interesting aspect of this analysis is that the load unbalance causes in turn an unbalance in the power channels. This aspect is only visible through the analysis performed using PV, and it is not possible by computing the instantaneous power using the measured voltage and current at the load terminals. Furthermore, it can be seen how the power flows through the channels, leading to an exchange between *R* and the source through every separated channel, and not an exchange between channels as suggested by^13^ or^40^. The need for a (real) compensating device to balance the system shows that this is a real and not fictitious effect. Although line *b* carries no current at all, the surrounding electric field is disturbed, and therefore the PV is influenced as well. This reality claim is equally valid both for the 3D case analyzed by^11^ and the 2D analogy presented hereby.

### Restoration of symmetry

One may now wonder what it will take to bring the system back to three-phase symmetry, i.e. to get the line currents to behave in a balanced way but deliver the same active power as before. Recall that a balanced system has currents as described in (15). However, we can even go a step further and set the conditions for an equivalent load to behave as a pure resistor consuming the same power as in the unbalanced case (Steinmetz compensator). This example has already been studied in^13^ using a simplified 3D model, which basically coincides with the one presented here. The currents once the symmetry is restored are

25 $$\begin{aligned} \begin{aligned} i_a(t)&=\sqrt{2}I\cos \omega t \\ i_b(t)&=\sqrt{2}I\cos (\omega t -120^{\circ })\\ i_c(t)&=\sqrt{2}I\cos (\omega t +120^{\circ })\\ \end{aligned} \end{aligned}$$

where $I=V/R$. For this new situation, the electric fields do not change as the voltages remain unchanged, but the magnetic fields do change according to the new currents. Recalling equation (11), the new values are

26 $$\begin{aligned} \begin{aligned} {\varvec{H}}_{1}&=i_a(t) \varvec{\sigma }_{yx} \\ {\varvec{H}}_{2}&=i_c(t) \varvec{\sigma }_{xy} \end{aligned} \end{aligned}$$

so that the new PVs are

27 $$\begin{aligned} \begin{aligned} {\varvec{s}}_{1}&={\varvec{H}}_1{\varvec{e}}_1=i_a\varvec{\sigma }_{yx} \frac{v_{ba}}{d_{1}} \varvec{\sigma }_{y}=\frac{v_{ab}i_a}{d_{1}} \varvec{\sigma }_{x}\\ {\varvec{s}}_{2}&={\varvec{H}}_2{\varvec{e}}_2=i_c\varvec{\sigma }_{xy} \frac{v_{cb}}{d_{2}} \varvec{\sigma }_{y}=\frac{v_{cb}i_c}{d_{2}} \varvec{\sigma }_{x}\\ \end{aligned} \end{aligned}$$

Using (12), the instantaneous powers associated with each channel are found as

28 $$\begin{aligned} \begin{aligned} p_1(t)&=v_{ab}i_a=\frac{{3}V^2}{R} \left[ \frac{1}{\sqrt{3}}\cos (2 \omega t + 30^{\circ }) + \frac{1}{2}\right] \\ p_2(t)&=v_{cb}i_c=\frac{{3}V^2}{R} \left[ -\frac{1}{\sqrt{3}} \cos (2 \omega t + 30^{\circ }) + \frac{1}{2}\right] \end{aligned} \end{aligned}$$

**Figure 5:**
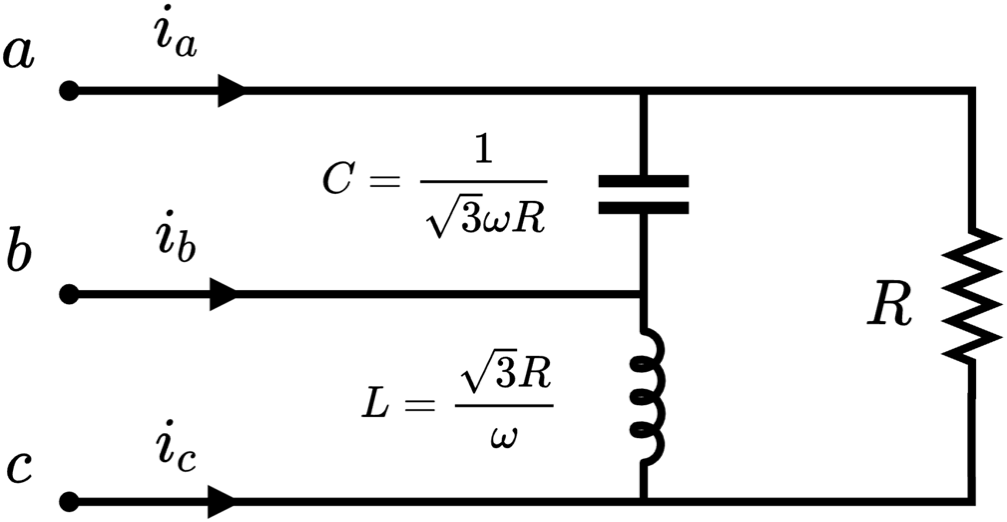
Steinmetz compensator. An inductor and a capacitor are added to the resistor in Fig. 3 to restore the symmetry in the currents.

Now we can see that the power flowing through each channel has an oscillating part that cancels out with that of the other channel, while there is still a constant component. The sum of the two channels is

29 $$\begin{aligned} p(t)=p_1(t)+p_2(t)=\frac{3V^2}{R} \end{aligned}$$

which is the traditional expression of the instantaneous power for a purely resistive balanced three-phase system. Note that the above result is not time-dependent. Once again it suggests that the power unbalance stems from a mismatch in channels 1 and 2. If we can achieve a setting that globally cancels the oscillating terms of the channels, then our system becomes balanced. What can provide the instantaneous power needed to restore symmetry? Steinmetz has already shown that this can be achieved using a capacitor and an inductor connected between phases *a* - *b* and *b* - *c*, as shown in Fig. 5. It can be easily proved that the value for the capacitor is $C=1/(\sqrt{3}\omega R)$ and for the inductor is $L=\sqrt{3}R/\omega$.

**Figure 6:**
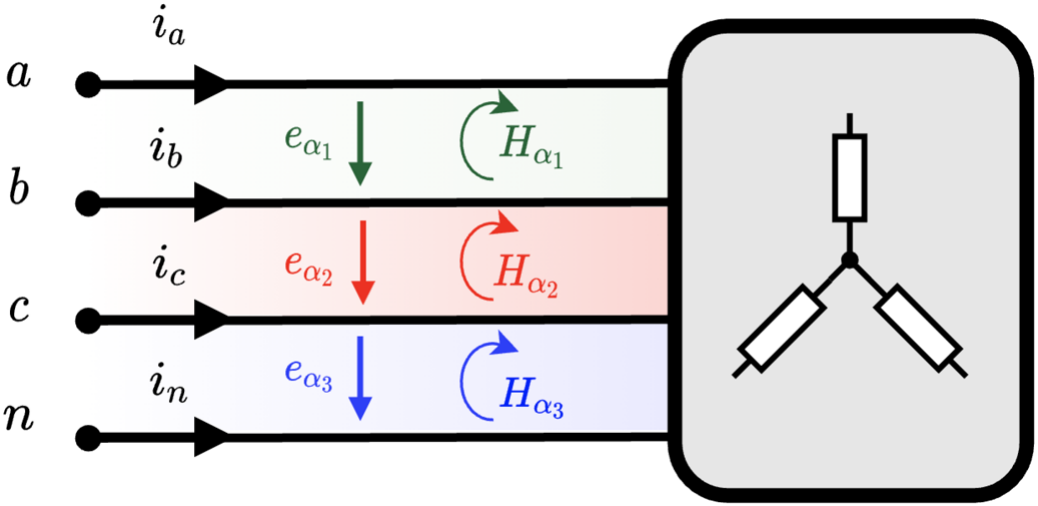
Generic Four-wire three-phase circuit. Three different power channels are formed (green, red and blue).

### Case of study: three-phase and four-wire load and symmetric voltage source

Let us now consider a three-phase four-wire system, i.e. 3 phases plus neutral as shown in Fig. 6. This configuration usually poses some challenges for very well-accepted power theories^41^ as they make assumptions that do not match the physical reality of the problem, especially under unbalanced configurations. Unlike the three-wire case, up to three different channels or regions can now be formed through which power can flow: $\alpha _1$, $\alpha _2$ and $\alpha _3$ (see Fig. 6). According to the way the load is configured internally, we can range from a completely balanced system to an unbalanced one, similar to that shown in Fig. 3.

Since the balanced situation implies that no current flows through the neutral, this scenario will be equivalent to the 3-wire circuit, so we are going to focus on a highly unbalanced system, consisting of a single resistor connected between phase and neutral. The physical position of the neutral will be varied, which will give rise to the formation of the above-mentioned different channels.

**Figure 7:**
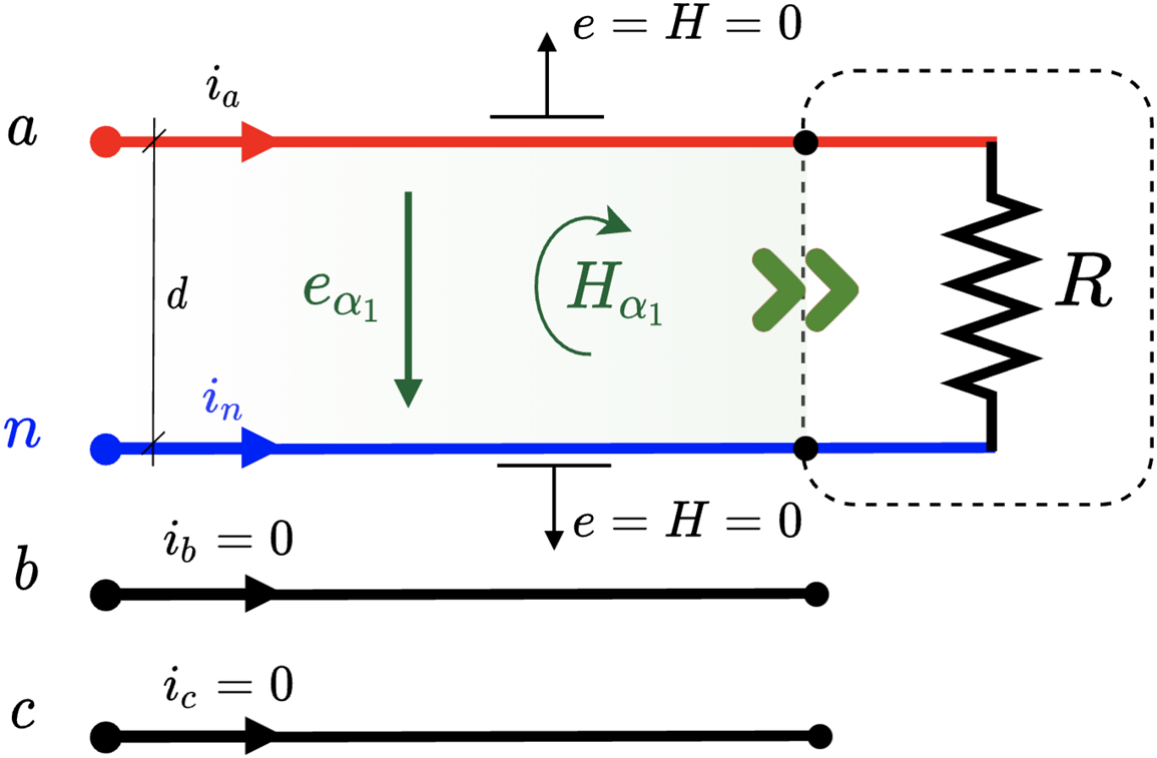
4-wire unbalanced 3-phase circuit with resistor between line *a* and neutral *n*.

#### Unbalanced system—1 channel

This configuration corresponds to the circuit in Fig. 7. Note that this configuration is the same as in a single-phase system. The magnetic and electric fields outside the channel are zero. Inside the channel the values are

30 $$\begin{aligned} \begin{aligned} {\varvec{e}}_{\alpha }&= -\frac{v_a}{d}\varvec{\sigma }_y \\ {\varvec{H}}_{\alpha }&= i_a\varvec{\sigma }_{yx}=\frac{v_a}{R}\varvec{\sigma }_{yx} \end{aligned} \end{aligned}$$

So the PV is computed as

31 $$\begin{aligned} {\varvec{s}}_{\alpha }={\varvec{H}}_{\alpha }{\varvec{e}}_{\alpha }=-\frac{v_a}{R}\varvec{\sigma }_{yx} \frac{v_{a}}{d_{}} \varvec{\sigma }_{y}=\frac{v^2_{a}}{Rd} \varvec{\sigma }_{x} \end{aligned}$$

and so the instantaneous power

32 $$\begin{aligned} p(t)=p_{\alpha }(t)={\varvec{s}}_{\alpha }\cdot d \varvec{\sigma }_x=\frac{v^2_{a}}{R} \end{aligned}$$

This result coincides with that expected by applying Ohm’s or Joule’s law directly to the resistor as a function of the voltage between its terminals. Note that the above results are valid for any permutation of voltage values $v_a$, $v_b$, or $v_c$ at the phase terminal of the $\alpha$ channel. Because there is only one power channel (as in single-phase circuits), there is little more to add regarding the way the power enters the load. This is not the case if the neutral is moved to another position as discussed below.

**Figure 8:**
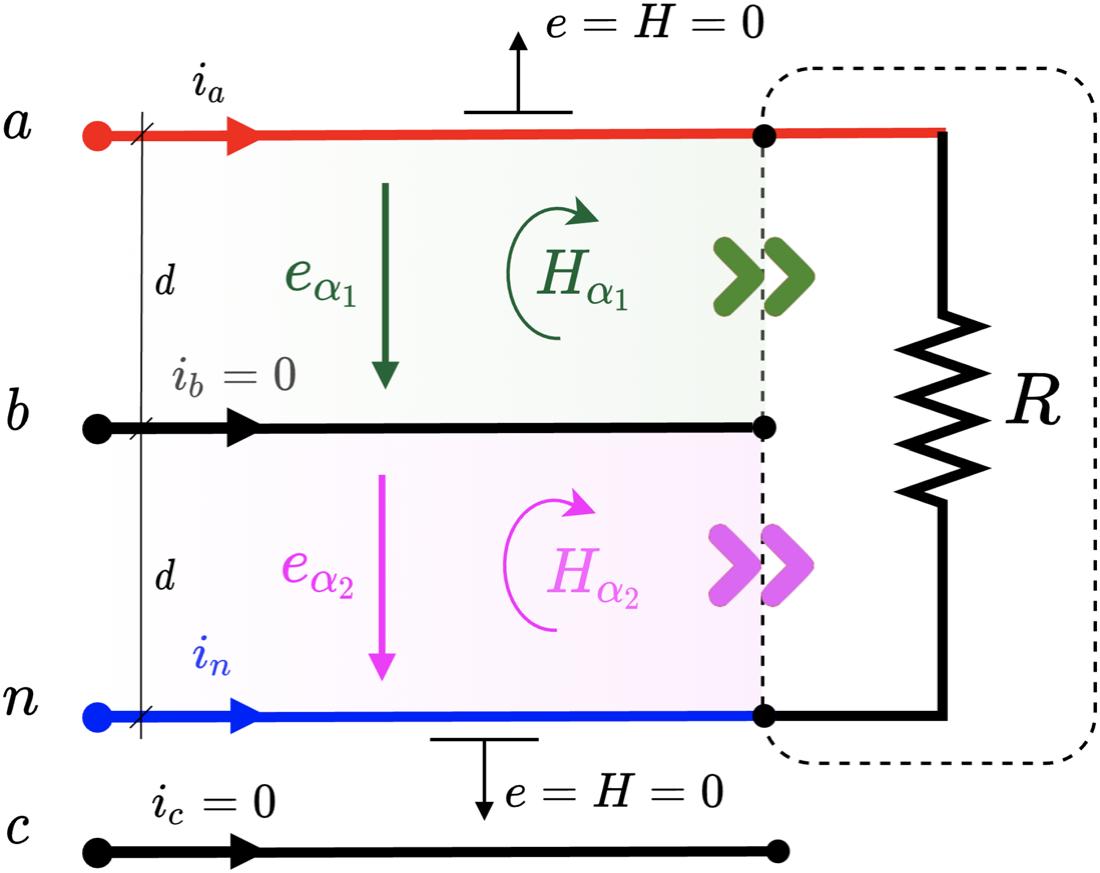
4-wire unbalanced 3-phase circuit with resistor between line *a* and neutral *n* and line *b* in between.

#### Unbalanced system—2 channels

This configuration corresponds to the one shown in Fig. 8. Now two channels $\alpha _1$ and $\alpha _2$ appear. Within these channels, the values of the fields are

33 $$\begin{aligned} \begin{aligned} {\varvec{e}}_{\alpha _1}&= -\frac{v_a-v_b}{d}\varvec{\sigma }_y \\ {\varvec{e}}_{\alpha _2}&= -\frac{v_b}{d}\varvec{\sigma }_y \\ {\varvec{H}}_{\alpha _1}&= {\varvec{H}}_{\alpha _2}=i_a\varvec{\sigma }_{yx}=\frac{v_a}{R}\varvec{\sigma }_{yx} \end{aligned} \end{aligned}$$

Thus, the PV can be computed as

34 $$\begin{aligned} \begin{aligned} {\varvec{s}}_{\alpha _1}&={\varvec{H}}_{\alpha _1}{\varvec{e}}_{\alpha _1}=-\frac{v_{a}}{R}\varvec{\sigma }_{yx} \frac{v_{ab}}{d_{}} \varvec{\sigma }_{y}=\frac{v_{a}v_{ab}}{Rd} \varvec{\sigma }_{x}\\ {\varvec{s}}_{\alpha _2}&={\varvec{H}}_{\alpha _2}{\varvec{e}}_{\alpha _2}=-\frac{v_a}{R}\varvec{\sigma }_{yx} \frac{v_{b}}{d_{}} \varvec{\sigma }_{y}=\frac{v_{a}v_b}{Rd} \varvec{\sigma }_{x} \end{aligned} \end{aligned}$$

and the instantaneous power is

35 $$\begin{aligned} p(t)=p_{\alpha _1}(t)+p_{\alpha _2}(t)={\varvec{s}}_{\alpha _1}\cdot d \varvec{\sigma }_x+{\varvec{s}}_{\alpha _2}\cdot d \varvec{\sigma }_x=\frac{v^2_{a}}{R} \end{aligned}$$

which again coincides with the expected value. Now the power flows through channels $\alpha _1$ and $\alpha _2$ although in an asymmetrical way. For example, for a sinusoidal power supply, as given in (14), the instantaneous power through channels $\alpha _1$ y $\alpha _2$ is

36 $$\begin{aligned} \begin{aligned} p_{\alpha _1}(t)&=\frac{v_a v_{ab}}{R}=\frac{V^2}{R} \left[ \sqrt{3}\cos (2 \omega t + 30^{\circ }) + \frac{3}{2}\right] \\ p_{\alpha _2}(t)&=\frac{v_av_{b}}{R}=\frac{V^2}{R} \left[ \cos (2 \omega t - 120^{\circ }) - \frac{1}{2}\right] \end{aligned} \end{aligned}$$

We see that there is a flow of negative active power through the $\alpha _2$ channel, while the $\alpha _1$ channel provides an additional 50% of active power to that consumed by the load as a whole.

**Figure 9:**
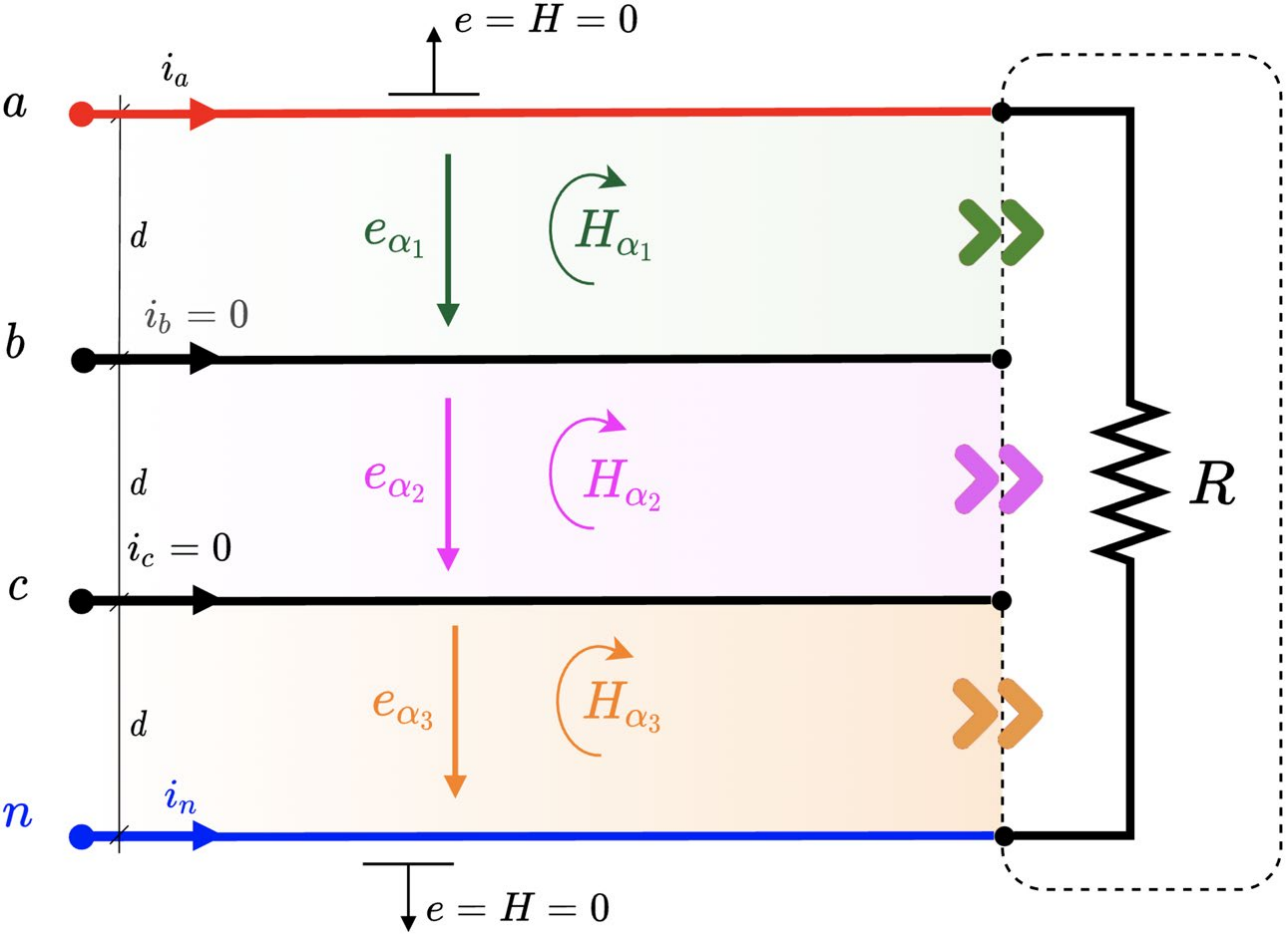
4-wire unbalanced 3-phase circuit with resistor between line *a* and neutral *n* with 2 phases in between.

#### Unbalanced system—3 channels

Finally, the case where the neutral is placed furthest away from the active phase is analyzed. This configuration corresponds to the one shown in Fig. 9. Now, three channels $\alpha _1$, $\alpha _2$ and $\alpha _3$ appear. Within these channels the values of the fields are

37 $$\begin{aligned} \begin{aligned} {\varvec{e}}_{\alpha _1}&= -\frac{v_a-v_b}{d}\varvec{\sigma }_y \\ {\varvec{e}}_{\alpha _2}&= -\frac{v_b-v_c}{d}\varvec{\sigma }_y \\ {\varvec{e}}_{\alpha _3}&= -\frac{v_c}{d}\varvec{\sigma }_y \\ {\varvec{H}}_{\alpha _1}&= {\varvec{H}}_{\alpha _2}={\varvec{H}}_{\alpha _3}=i_a\varvec{\sigma }_{yx}=\frac{v_a}{R}\varvec{\sigma }_{yx} \end{aligned} \end{aligned}$$

The Poynting vector for every channel is

38 $$\begin{aligned} \begin{aligned} {\varvec{s}}_{\alpha _1}&={\varvec{H}}_{\alpha _1}{\varvec{e}}_{\alpha _1}=-\frac{v_{a}}{R}\varvec{\sigma }_{yx} \frac{v_{ab}}{d_{}} \varvec{\sigma }_{y}=\frac{v_{a}v_{ab}}{Rd} \varvec{\sigma }_{x}\\ {\varvec{s}}_{\alpha _2}&={\varvec{H}}_{\alpha _2}{\varvec{e}}_{\alpha _2}=-\frac{v_a}{R}\varvec{\sigma }_{yx} \frac{v_{bc}}{d_{}} \varvec{\sigma }_{y}=\frac{v_{a}v_{bc}}{Rd} \varvec{\sigma }_{x}\\ {\varvec{s}}_{\alpha _3}&={\varvec{H}}_{\alpha _3}{\varvec{e}}_{\alpha _3}=-\frac{v_a}{R}\varvec{\sigma }_{yx} \frac{v_{c}}{d_{}} \varvec{\sigma }_{y}=\frac{v_{a}v_c}{Rd} \varvec{\sigma }_{x} \end{aligned} \end{aligned}$$

and the instantaneous power is

39 $$\begin{aligned} \begin{aligned} p(t)&=p_{\alpha _1}(t)+p_{\alpha _2}(t)+p_{\alpha _3}(t)\\&={\varvec{s}}_{\alpha _1}\cdot d \varvec{\sigma }_x+{\varvec{s}}_{\alpha _2}\cdot d \varvec{\sigma }_x+{\varvec{s}}_{\alpha _3}\cdot d \varvec{\sigma }_x=\frac{v^2_{a}}{R} \end{aligned} \end{aligned}$$

which again coincides with the expected value. For a sinusoidal voltage, we get

40 $$\begin{aligned} \begin{aligned} p_{\alpha _1}(t)&=\frac{v_a v_{ab}}{R}=\frac{V^2}{R} \left[ \sqrt{3}\cos (2 \omega t + 30^{\circ }) + \frac{3}{2}\right] \\ p_{\alpha _2}(t)&=\frac{v_a v_{bc}}{R}=\frac{V^2}{R} \left[ \sqrt{3}\cos (2 \omega t - 90^{\circ }) \right] \\ p_{\alpha _3}(t)&=\frac{v_av_{c}}{R}=\frac{V^2}{R} \left[ \cos (2 \omega t + 120^{\circ }) - \frac{1}{2}\right] \end{aligned} \end{aligned}$$

The distribution of power per channel varies concerning the previous case. Interestingly, the central channel $\alpha _2$ does not transmit active power, but only oscillating power. Similar to the previous case, channel $\alpha _1$ carries more active power than is consumed by the load, while channel $\alpha _3$ forwards power to the source, so that the sum of the power of the 3 channels coincides with the total instantaneous power.

As previously discussed in the 2D analogy of the Steinmetz compensator, in these examples it has been considered that not all the wires are current-carrying, even though they are all energised, so this does not preclude the conclusion that these are purely fictitious effects.

## Conclusions

In this work, the power flow problem in three-phase three- and four-wire circuits has been formulated using the basic laws of electromagnetism expressed mathematically using geometric algebra (GA) for theoretical two-dimensional (planar) circuits. The magnetic field $\varvec{H}$ is presented as a bivector embedded in the 2D plane so that the geometric product between the electric field $\varvec{e}$ and $\varvec{H}$ can be computed to obtain the Poynting Vector in the so-called *power channels* ($\alpha _k$). The instantaneous power is completely determined by adding the power of every channel as the product of the PV and the width of the channel $\alpha _k$. The results are consistent with the usual definition of instantaneous power. The use cases analyzed show how power flows through the channels even when there are no current-carrying conductors. This fact is revealing as it explains how the oscillating terms of the power flow play out. For balanced systems, the oscillating terms cancel each other out when all channels are summed. In contrast, for unbalanced circuits, this does not happen. All formed channels show oscillating terms. The compensation turns out to be easy and generalizable to *m* wires. The PV is shown to be the key physical tool to fully understand the power transport phenomena.

## Methods

This section is intended to allow readers to reproduce our work if necessary. Due to the main theoretical nature of our present contribution, the results can be checked simply by making the same calculations that we provide, but instead of using Geometric Algebra in two dimensions resorting to the common Gibbs Vector Algebra in three dimensions. The absolute coincidence of both calculations is a proof for the suitability of GA to provide straightforward computations with a clear geometrical meaning.

## Supplementary Information

Supplementary Information.

## Data Availability

The datasets used and/or analysed during the current study are available from the corresponding author upon reasonable request. Please, contact the main author Francisco G. Montoya (pagilm@ual.es).
