# Author Correction: New mathematical model based on geometric algebra for physical power flow in theoretical two-dimensional multi-phase power circuits

**DOI:** 10.1038/s41598-023-29836-x

**Published:** 2023-02-15

**Authors:** Francisco G. Montoya, Xabier Prado, Francisco M. Arrabal-Campos, Alfredo Alcayde, Jorge Mira

**Affiliations:** 1 Department of Engineering, University of Almeria, 04120 Almeria, Spain; 2 Departamento de Física Aplicada and iMATUS, Universidade de Santiago de Compostela, 15782 Santiago de Compostela, Spain

Correction to: *Scientific Reports* 10.1038/s41598-023-28052-x, published online 20 January 2023

The original version of this Article contained an error in the Applications and examples section, where an example description was placed incorrectly under the subheading ‘Three‑phase and three‑wire balanced load and symmetric voltage supply.’

As a result, the following description was now placed under the correct subheading ‘Three‑phase and three‑wire unbalanced resistor load and symmetric voltage supply.’:

“One may wonder how these channels may be formed since no current is flowing through line *b*. The explanation is simple. Although no current flows through *b*, the voltage of phase *b* is not null and therefore it affects the electric field $\varvec{e}_1$ and $\varvec{e}_2$, and hence the PV of the channels $\alpha_1$ and $\alpha_2$. Interestingly enough, line *b* is some kind of “electrical wall”.”

The original Article has been corrected.

