# Supplementary material for: New mathematical model based on geometric algebra for physical power flow in theoretical two-dimensional multi-phase power circuits
Source: Sci Rep. 2023 Jan 20;13:1128. doi: 10.1038/s41598-023-28052-x (PMC9860059; doi:10.1038/s41598-023-28052-x)
Supplement: Supplementary file 1 — Supplementary Information. [file 41598_2023_28052_MOESM1_ESM.pdf]

Supplemental Material

immediate

APPENDIX A - Basic Concepts of GA

The use and management of vectors can be extended using Geometric Algebra (GA). Crucial to this is the geometric product, an operation between two vectors $\boldsymbol{a}$ and $\boldsymbol{b}$ that produces a twofold result:

$$\boldsymbol{ab} = \boldsymbol{a} \cdot \boldsymbol{b} + \boldsymbol{a} \wedge \boldsymbol{b} \tag{A.1}$$

where $\boldsymbol{a} \cdot \boldsymbol{b}$ is a scalar (inner product), while $\boldsymbol{a} \wedge \boldsymbol{b}$ (outer product) is a new entity known as a bivector. This geometric product is invertible, which is a very useful property for algebraic calculations. This procedure allows us to construct new objects of increasing dimensionality (or degree), called blades. These blades of different degree can be added together to form multivectors. The set of multivectors in a space of a certain dimension has the structure of an algebra, and a basis can be established consisting of elements of all the multivectors considered. In the Euclidean geometric space, it is possible to construct an orthonormal vector basis formed by the elements $\boldsymbol{\sigma}_1, \boldsymbol{\sigma}_2, \ldots, \boldsymbol{\sigma}_k$ with a positive signature $\boldsymbol{\sigma}_i^2 = 1$. It is also possible to construct a bivectorial basis:

$$\boldsymbol{\sigma}_{ij} = \boldsymbol{\sigma}_i \boldsymbol{\sigma}_j = \boldsymbol{\sigma}_i \wedge \boldsymbol{\sigma}_j = -\boldsymbol{\sigma}_{ji} \tag{A.2}$$

with a negative signature $\boldsymbol{\sigma}_{ij}^2 = -1$. In two dimensions (Euclidean plane), a general basis of the geometric algebra can be $\boldsymbol{\sigma} = \{1, \boldsymbol{\sigma}_x, \boldsymbol{\sigma}_y, \boldsymbol{\sigma}_{xy}\}$ To understand the geometric product between a vector and a bivector from a purely geometric point of view, we turn to the elements of the respective bases:

$$\begin{aligned} \boldsymbol{\sigma}_x \boldsymbol{\sigma}_{xy} &= \boldsymbol{\sigma}_y = -\boldsymbol{\sigma}_{xy} \boldsymbol{\sigma}_x \\ \boldsymbol{\sigma}_y \boldsymbol{\sigma}_{xy} &= -\boldsymbol{\sigma}_x = -\boldsymbol{\sigma}_{xy} \boldsymbol{\sigma}_y \end{aligned} \tag{A.3}$$

We can see in Fig. A1 that it is possible to visually understand this product if we consider that the bivector $\boldsymbol{\sigma}_{xy}$ acts on a vector by rotating it by 90º. Depending on the side it multiplies by, the rotation can be positive or negative. This property is similar to the imaginary unit j in the algebra of complex numbers. Recall that such an algebra is isomorphic to the even GA algebra of dimension two, i.e., $\mathscr{G}^+(2,0)$.

APPENDIX B - Maxwell's equations in GA

To formulate Maxwell's equations in GA terms, it is necessary to define a new operator that includes the time derivative and the nabla operator. For the traditional three dimensional space, it reads

$$\begin{aligned} \nabla &= \partial_0 + \boldsymbol{\nabla} \\ \partial_0 &= \partial_t = \frac{\partial}{\partial_t} \\ \boldsymbol{\nabla} &= \partial_x \boldsymbol{\sigma}_x + \partial_y \boldsymbol{\sigma}_y + \partial_z \boldsymbol{\sigma}_z = \partial_k \boldsymbol{\sigma}_k = \frac{\partial}{\partial x_k} \boldsymbol{\sigma}_k \end{aligned} \tag{B.1}$$

On the other hand, a multivector $\boldsymbol{J}$ can be defined where the scalar part is the charge density, and the vector part equals the current density.

$$\begin{aligned} \boldsymbol{J} &= \rho - \boldsymbol{j} \\ \boldsymbol{j} &= j_x \boldsymbol{\sigma}_x + j_y \boldsymbol{\sigma}_y + j_z \boldsymbol{\sigma}_z = j_k \boldsymbol{\sigma}_k \end{aligned} \tag{B.2}$$

The negative sign of $\boldsymbol{j}$ is due to the transition between Euclidean space (with all positive signatures) and spacetime (where the time signature is opposite to the space signature). On the other hand, we define the multivector electromagnetic field $\boldsymbol{F}$ as

$$\boldsymbol{F} = \boldsymbol{e} + \boldsymbol{H} \tag{B.3}$$

where

$$\begin{aligned} \boldsymbol{e} &= e_x \boldsymbol{\sigma}_x + e_y \boldsymbol{\sigma}_y + e_z \boldsymbol{\sigma}_z = e_k \boldsymbol{\sigma}_k \\ \boldsymbol{H} &= H_z \boldsymbol{\sigma}_{xy} + H_x \boldsymbol{\sigma}_{yz} + H_y \boldsymbol{\sigma}_{zx} \end{aligned} \tag{B.4}$$

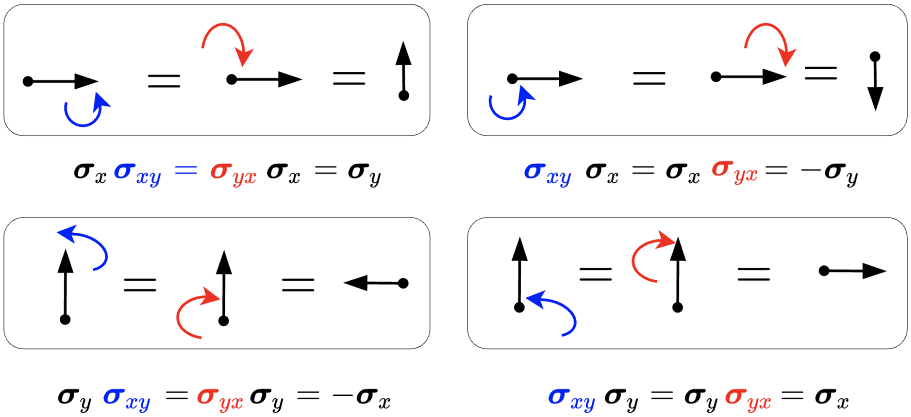

Figure A1. Visualisation of the geometric product between a vector and a bivector in the Euclidean plane. The vector undergoes a rotation of $\pm 90°$ due to the bivector $\boldsymbol{\sigma}_{xy} = -\boldsymbol{\sigma}_{yx}$ depending on the multiplication side. As a rule of thumb, right multiplication acts on the end of the vector while left multiplication acts on the origin of the vector.

Thus, Maxwell's equations can be written as

$$(\partial_t + \boldsymbol{\nabla})(\boldsymbol{e} + \boldsymbol{H}) = \rho - \boldsymbol{j} \tag{B.5}$$

In the general (3D) case, these equations can be separated into four different multivector terms corresponding to the four equations forming Maxwell's laws:

$$\begin{aligned} \boldsymbol{\nabla} \cdot \boldsymbol{e} &= \rho && \text{(Gauss's Law)} \\ \boldsymbol{\nabla} \boldsymbol{H} &= -\boldsymbol{j} - \partial_t \boldsymbol{e} && \text{(Ampère's Law)} \\ \boldsymbol{\nabla} \wedge \boldsymbol{e} &= -\partial_t \boldsymbol{H} && \text{(Faraday's Law)} \\ \boldsymbol{\nabla} \wedge \boldsymbol{H} &= 0 && \text{(Gauss's Law for MF)} \end{aligned} \tag{B.6}$$

In a two-dimensional space, the last equation is no longer applicable. The physical variables involved can be described in their components as follows

$$\begin{aligned} \boldsymbol{j} &= j_x \boldsymbol{\sigma}_x + j_y \boldsymbol{\sigma}_y \\ \boldsymbol{e} &= e_x \boldsymbol{\sigma}_x + e_y \boldsymbol{\sigma}_y \\ \boldsymbol{H} &= H \boldsymbol{\sigma}_{xy} \end{aligned} \tag{B.7}$$

Thus, with the above notation, 2D Maxwell's laws look like this:

$$\begin{aligned} \partial_x e_x + \partial_y e_y &= \rho && \text{(Gauss)} \\ \partial_y H &= j_x + \partial_t e_x && \text{(Ampère-x)} \\ -\partial_x H &= j_y + \partial_t e_y && \text{(Ampère-y)} \\ \partial_x e_y - \partial_y e_x &= -\partial_t H && \text{(Faraday)} \end{aligned} \tag{B.8}$$

To establish such expressions, we have taken into account the geometric products defined in (A.3). The above are differential expressions, which can be integrated using the fundamental theorem of geometric calculus[?]

$$\int_{\mathscr{V}} \boldsymbol{\nabla} \boldsymbol{M} \, d\boldsymbol{\tau} = \oint_{\mathscr{S}} \boldsymbol{M} \cdot d\boldsymbol{s} \tag{B.9}$$

where $\boldsymbol{M}$ is a general multivector, $\mathscr{V}$ is the enclosing hyper-volume and $\mathscr{S}$ is the boundary hyper-surface of $\mathscr{V}$.

In three-dimensional Euclidean space, $\mathscr{V}$ would be a certain volume bounded by a surface $\mathscr{S}$, while in a plane space, $\mathscr{V}$ would be a surface bounded by a closed curve $\mathscr{S}$. As a limiting case, for a one-dimensional world, $\mathscr{V}$ would be a segment and $\mathscr{S}$ the pair of points bounding it.
